# Mathematical Model for Coronavirus Disease 2019 (COVID-19) Containing Isolation Class

**DOI:** 10.1155/2020/3452402

**Published:** 2020-06-25

**Authors:** Anwar Zeb, Ebraheem Alzahrani, Vedat Suat Erturk, Gul Zaman

**Affiliations:** ^1^Department of Mathematics, COMSATS University Islamabad, Abbottabad Campus, Abbottabad 22060, Khyber Pakhtunkhwa, Pakistan; ^2^Department of Mathematics, Faculty of Science, King Abdulaziz University, P.O. Box 80203, Jeddah 21589, Saudi Arabia; ^3^Department of Mathematics, Faculty of Arts and Sciences, Ondokuz Mayis University, 55139 Samsun, Turkey; ^4^Department of Mathematics, University of Malakand, Chakdara 18000, Dir (Lower), Khyber Pakhtunkhwa, Pakistan

## Abstract

The deadly coronavirus continues to spread across the globe, and mathematical models can be used to show suspected, recovered, and deceased coronavirus patients, as well as how many people have been tested. Researchers still do not know definitively whether surviving a COVID-19 infection means you gain long-lasting immunity and, if so, for how long? In order to understand, we think that this study may lead to better guessing the spread of this pandemic in future. We develop a mathematical model to present the dynamical behavior of COVID-19 infection by incorporating isolation class. First, the formulation of model is proposed; then, positivity of the model is discussed. The local stability and global stability of proposed model are presented, which depended on the basic reproductive. For the numerical solution of the proposed model, the nonstandard finite difference (NSFD) scheme and Runge-Kutta fourth order method are used. Finally, some graphical results are presented. Our findings show that human to human contact is the potential cause of outbreaks of COVID-19. Therefore, isolation of the infected human overall can reduce the risk of future COVID-19 spread.

## 1. Introduction

Mathematical models are useful to understand the behavior of an infection when it enters a community and investigate under which conditions it will be wiped out or continued. Currently, COVID-19 is of great concern to researches, governments, and all people because of the high rate of the infection spread and the significant number of deaths that occurred. In December 2019, coronavirus first reported in Wuhan, China, is an infectious disease caused by a newly discovered coronavirus. The virus that causes COVID-19 is mainly transmitted through droplets generated when an infected person coughs, sneezes, or exhales. These droplets are too heavy to hang in the air and quickly fall on floors or surfaces. Coronavirus-confirmed cases reached nearly four million in 187 countries, and approximately 295,000 people have lost their lives due to this virus.

**Figure 1:**
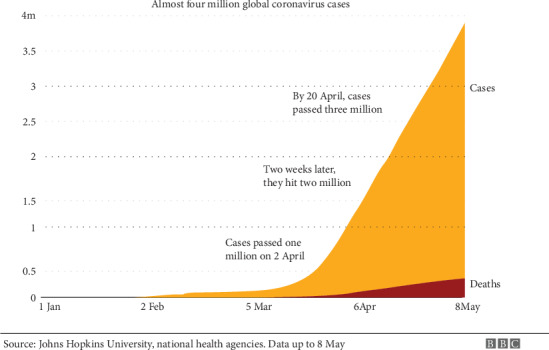
BBC News up to 8 May [1].

According to figures collated by Johns Hopkins University, the largest cases occurred at the US. Noting that more than 77,000 deaths happened, it also has the world's highest death toll (see the Figure 1) [1].

Researchers have been tracking the spread of the virus, have mobilized to speed innovative diagnostics, and are working on a number of vaccines to protect against COVID-19. Cao et al. [2, 3] studied the clinical features of coronavirus and discussed the short-term outcomes of 18 patients and 102 patients with COVID-19 in intensive care units. For the demographic details of 102 patients, see Table 1 [3]. Coronaviruses are typically transmitted from person to person through respiratory droplets and close contact. The majority of the transmission is happening through respiratory droplets that we may inhale from close contact with one another [4]. A modified SIR epidemic model is presented in [4] to project the actual number of infected cases and the specific burdens on isolation wards and intensive care units. Nesteruk [5] developed an *SIR* (susceptible, infected, and recovered) epidemic model and discussed statistically the parameters used in the proposed model and showed how to control this infection.

Unfortunately, the number of coronavirus victims is expected to be much higher than that predicted on February 10, 2020, since 12289 new cases (not previously included in official counts) have been added two days later. Further research should focus on updating the predictions with the use of up-to-date data and using more complicated mathematical models. Currently, there are no licensed vaccines or therapeutic agents for coronavirus prevention or treatment though research studies into potential antivirals and vaccine candidates are underway in a number of countries. Vaccine testing, development, and distribution are typically a much longer process than drug development, and it is not likely that a vaccine for COVID-19 will be ready before 2021 at the earliest. The virus can easily spread in dense places. Social distancing or low contact rate refers to measures that are taken to increase the physical space between people to slow down the spread of the virus. Batista [6] studied the logistic growth regression model which is used for the estimation of the final size of the coronavirus epidemic. Several researchers developed different models of COVID-19 and studied dynamical behavior (see for instance [4–7]).

From the above discussion, it was concluded that human to human contact is the potential cause of outbreaks of COVID-19. Therefore, isolation of the infected human overall can reduce the risk of future COVID-19 spread. In order to do this, we divided the total population into five compartments: susceptible, exposed, infected, isolated, and recovered from the disease. This study will lead to the mathematical model formulation in which the interaction of the exposed population and infected population occurred to the susceptible populations. The infected individuals, the individuals showing no symptoms apparently but have the disease in weak form inside their bodies, must be sent to isolated class in different rates. The local stability and global stability of model is discussed, by using the approach of the basic reproductive. For the numerical solution of the proposed model, the nonstandard finite difference (NSFD) scheme and Runge-Kutta fourth order method are used. Finally, some graphical results are presented. Our findings show that human to human contact is the potential cause of outbreaks of COVID-19.

The paper is organised as follows: Section 2 is related to the model formulation keeping in mind the assumptions that exposed and infected individuals are making contacts with susceptible individuals at the same rate.  Section 3 is concerned with the local stability and existence of positive equilibrium solution. Some numerical simulations are executed to illustrate the analytical results in Section 4. Finally, conclusions are presented in Section 5.

## 2. Model Formulation

In this section, we develop the mathematical model by taking into account the above assumptions.

(1) $$\begin{array}{c} \left\{\begin{array}{c} \frac{dS\left(t\right)}{dt}=A-\mu S\left(t\right)-\beta \left(N\right)S\left(t\right)\left(E\left(t\right)+I\left(t\right)\right),\\ \frac{dE\left(t\right)}{dt}=\beta \left(N\right)S\left(t\right)\left(E\left(t\right)+I\left(t\right)\right)-\pi E\left(t\right)-\left(\mu +\gamma \right)E\left(t\right),\\ \frac{dI\left(t\right)}{dt}=\pi E\left(t\right)-\sigma I\left(t\right)-\mu I\left(t\right),\\ \frac{dQ\left(t\right)}{dt}=\gamma E\left(t\right)+\sigma I\left(t\right)-\theta Q\left(t\right)-\mu Q\left(t\right),\\ \frac{dR\left(t\right)}{dt}=\theta Q\left(t\right)-\mu R\left(t\right), \end{array}\right. \end{array}$$

where the parameters and variables used are described in Table 1.

As the first four equations are independent of *R*(*t*), so omit without generality the last equation for *R*(*t*) and the modified system (1) becomes

(2) $$\begin{array}{c} \left\{\begin{array}{c} \frac{dS\left(t\right)}{dt}=A-\mu S\left(t\right)-\beta \left(N\right)S\left(t\right)\left(E\left(t\right)+I\left(t\right)\right),\\ \frac{dE\left(t\right)}{dt}=\beta \left(N\right)S\left(t\right)\left(E\left(t\right)+I\left(t\right)\right)-\pi E\left(t\right)-\left(\mu +\gamma \right)E\left(t\right),\\ \frac{dI\left(t\right)}{dt}=\pi E\left(t\right)-\sigma I\left(t\right)-\mu I\left(t\right),\\ \frac{dQ\left(t\right)}{dt}=\gamma E\left(t\right)+\sigma I\left(t\right)-\theta Q\left(t\right)-\mu Q\left(t\right). \end{array}\right. \end{array}$$

For system (2), let *N* = *A*/*μ*, *s* = *S*/*N*, *e* = *E*/*N*, *i* = *I*/*N*, and *q* = *Q*/*N*, and rescale the system (2) to get the normalized form

(3) $$\begin{array}{c} \frac{ds}{dt}=\mu -\mu s-\beta Ns\left(e+i\right), \end{array}$$

(4) $$\begin{array}{c} \frac{de}{dt}=\beta Ns\left(e+i\right)-\pi e-\left(\mu +\gamma \right)e, \end{array}$$

(5) $$\begin{array}{c} \frac{di}{dt}=\pi e-\sigma i-\mu i, \end{array}$$

(6) $$\begin{array}{c} \frac{dq}{dt}=\gamma e+\sigma i-\theta q-\mu q, \end{array}$$

with the initial conditions

(7) $$\begin{array}{c} s\left(0\right)=s_{0}\geq 0,\;e\left(0\right)=e_{0}\geq 0,\;i\left(0\right)=i_{0}\geq 0,\;q\left(0\right)=q_{o}\geq 0. \end{array}$$

In the remaining sections, we will discuss the local and global stability of the proposed model with initial conditions. First, a result is observed for the positivity and boundedness of the solution of system (3).

**Lemma 1 .**

Under the initial conditions (7), all the solutions (*s*(*t*), *e*(*t*), *i*(*t*), *q*(*t*)) of system (3) remain nonnegative for *t* ≥ 0

**Proof**

By the initial conditions (7), it was discovered that

(8) $$\begin{array}{c} \frac{ds}{dt}\left|{}_{s=0}=\mu \right.>0,\\ \frac{de}{dt}\left|{}_{e=0}=\beta Nsi\geq 0,\right.\\ \frac{di}{dt}\left|{}_{i=0}=\pi e\geq 0,\right.\\ \frac{dq}{dt}\left|{}_{q=0}=\gamma e+\sigma i\geq 0.\right. \end{array}$$

## 3. Local Stability and Existence of Positive Equilibrium Point

The existence of unique positive equilibrium and stability of system (3) depends on the basic reproductive number *ℜ*_0_ on free equilibrium point (FEP) *C*_0_, which is determined with the help of the next generation matrix method [8]. The free coronavirus equilibrium point is *C*_0_ = (1, 0, 0, 0).

Consider the following matrices for finding the basic reproductive number *ℜ*_0_:

(9) $$\begin{array}{c} F=\left(\begin{array}{c} \beta Ns\left(e+i\right)\\ 0 \end{array}\right),\\ V=\left(\begin{array}{c} \pi e+\left(\mu +\gamma \right)e\\ -\pi e+\sigma i+\mu i \end{array}\right). \end{array}$$

Now Jacobian of *F* and *V* at *C*_0_ are

(10) $$\begin{array}{c} F=\left(\begin{array}{cc} \beta N & \beta N\\ 0 & 0 \end{array}\right),\\ V=\left(\begin{array}{cc} \pi +\mu +\gamma & 0\\ -\pi & \sigma +\mu \end{array}\right). \end{array}$$

The dominant eigenvalue of *FV*^−1^ represents *ℜ*_0_ = *ρ*(*FV*^−1^), which is

(11) $$\begin{array}{c} R_{0}=\beta N\frac{\sigma +\mu +\pi }{\left(\pi +\mu +\gamma \right)\left(\sigma +\mu \right)}. \end{array}$$

**Theorem 1 .**

The system (3) is locally stable related to virus-free equilibrium point *C*_0_, *ℜ*_0_ < 1 and unstable if *ℜ*_0_ > 1.

**Proof**

For local stability at *C*_0_, the Jacobian of system (3) is

(12) $$\begin{array}{c} J=\left(E_{0}\right)=\left(\begin{array}{cccc} -\mu & -\beta N & -\beta N & 0\\ 0 & \beta N-\pi -\mu -\gamma & \beta N & 0\\ 0 & \pi & -\left(\sigma +\mu \right) & 0\\ 0 & \gamma & \sigma & -\left(\theta +\mu \right) \end{array}\right), \end{array}$$

which follows the eigenvalues , *λ*_2_ < 0, *λ*_3_ < 0, and *λ*_4_ < 0, if *ℜ*_0_ < 1. So the system (3) is locally stable if *ℜ*_0_ < 1 and unstable if *ℜ*_0_ > 1.

**Theorem 2 .**

There exists a unique positive virus equilibrium point *C*^∗^(*s*^∗^, *e*^∗^, *i*^∗^, *q*^∗^) for system (3), if *ℜ*_0_ > 1.

**Proof**

By letting the right hand sides of all equations of system (3) to zero, as

(13) $$\begin{array}{c} \mu -\mu s-\beta Ns\left(e+i\right)=0,\\ \beta Ns\left(e+i\right)-\pi e-\left(\mu +\gamma \right)e=0,\\ \pi e-\sigma i-\mu i=0,\\ \gamma e+\sigma i-\theta q-\mu q=0 \end{array}$$

implies that

(14) $$\begin{array}{c} s^{*}=\frac{1}{R_{0}},\\ e^{*}=\frac{\left(\sigma +\mu \right)}{\pi }i^{*},\\ i^{*}=\frac{\pi \mu \left(R_{0}-1\right)}{\beta N\left(\pi +\sigma +\mu \right)},\\ q^{*}=\frac{\gamma \left(\sigma +\mu \right)+\pi \sigma }{\pi \left(\theta +\mu \right)}i^{*}. \end{array}$$

From the value of *i*^∗^, it is obvious that all the values of (*s*^∗^, *e*^∗^, *q*^∗^) are positive if *ℜ*_0_ > 1.

**Theorem 3 .**

If *ℜ*_0_ < 1, then the system (3) is globally stable.

**Proof**

For the proof of this theorem, first, we construct the Lyapunov function *L* as

(15) $$\begin{array}{c} L=\left(e-e_{0}\right)+\frac{\beta N}{\sigma +\mu }\left(i-i_{0}\right). \end{array}$$

Differentiating equation (15) with respect to time and keeping the reality in mind that *ℜ*_0_ < 1 and 0 < *s* < 1, we obtained

(16) $$\begin{array}{c} L^{\prime }=\overset{´}{e}+\frac{\beta N}{\sigma +\mu }\overset{´}{i},\\ L^{\prime }=\beta Nse+\beta Nsi-\left(\pi +\mu +\gamma \right)e+\frac{\beta N}{\sigma +\mu }\left(\pi e\right)-\frac{\beta N}{\sigma +\mu }\left(\sigma +\mu \right)i,\\ L^{\prime }=\beta Nse+\beta Nsi-\left(\pi +\mu +\gamma \right)e+\frac{\beta N}{\sigma +\mu }\left(\pi e\right)-\beta Ni,\\ \leq \beta Ne-\left(\pi +\mu +\gamma \right)e+\frac{\beta N}{\sigma +\mu }\left(\pi e\right)=\left(R_{0}-1\right)e. \end{array}$$

Therefore, if *R*_0_ < 1, then *L*′ < 0, which implies that the system (3) is globally stable for *R*_0_ < 1.

## 4. Numerical Method and Results

The NSFD method is used for the numerical solution of the proposed model (3). Basically, NSFD is an iterative method in which we get closer to solution through iteration [9, 10]. Let nonstandard ODEs be given as follows:

(17) $$\begin{array}{c} y\prime_{k}=f\left[t,y_{1},y_{2},\cdots ,y_{n}\right], \end{array}$$

where *k* = 1, 2, ⋯, *n*, then, by the NFSD method

(18) $$\begin{array}{c} \begin{array}{c} y_{1}^{\prime }=\frac{y_{1},k+1-y_{1},k}{h},\\ y_{2}^{\prime }=\frac{y_{2},k+1-y_{2},k}{h},\\ \vdots \\ y_{n}^{\prime }=\frac{y_{n},k+1-y_{n},k}{h}. \end{array} \end{array}$$

Now, using the NSFD method for numerical solution of system (3), it follows that

(19) $$\begin{array}{c} s_{k+1}=\frac{h\mu +s_{k}}{\left(1+h\beta N_{k}i_{k}+h\beta N_{k}e_{k}+h\mu s_{k}+1\right)},\\ e_{k+1}=\frac{h\beta N_{k}s_{k+1}i_{k}+h\beta N_{k}s_{k+1}e_{k}+e_{k}}{1+h\left(\pi +\mu +\gamma \right)},\\ i_{k+1}=\frac{h\pi e_{k+1}+i_{k}}{1+h\left(\sigma +\mu \right)},\\ q_{k+1}=\frac{h\gamma e_{k+1}+h\sigma i_{k+1}+q_{k}}{1+h\left(\theta +\mu \right)}. \end{array}$$

We assume the parameters of the system (3) shown in Table 1 [4].

**Figure 2:**
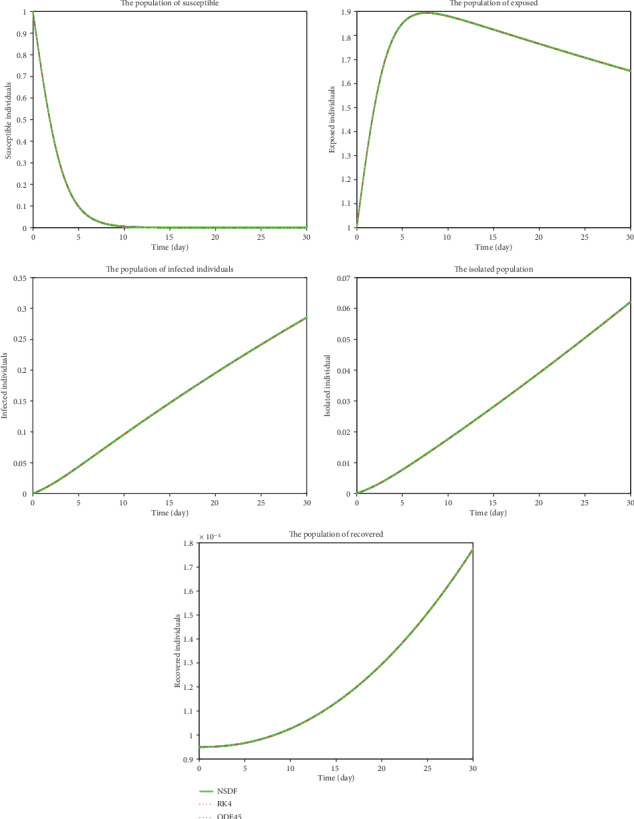
Plots present the susceptible, exposed, infected, isolated, and recovered population when *ℜ*_0_ > 1.

**Figure 3:**
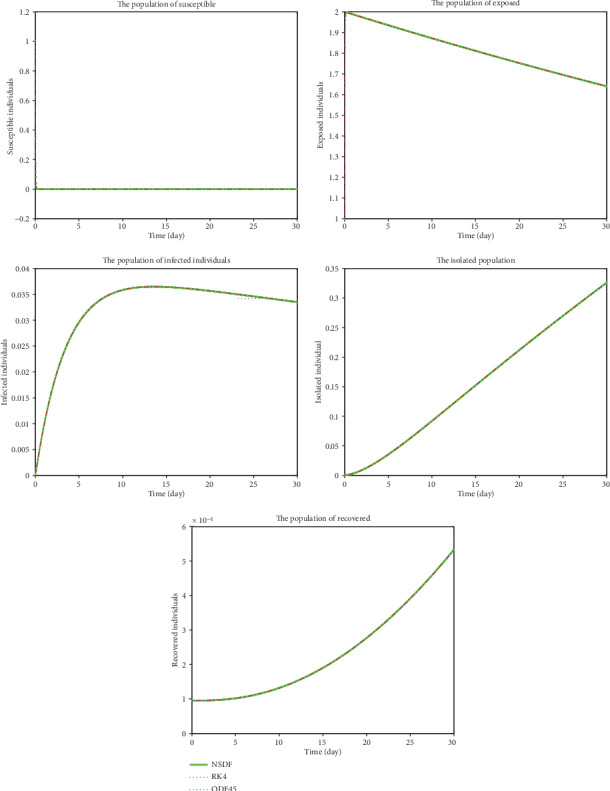
Plots present the susceptible, exposed, infected, isolated and recovered population when *ℜ*_0_ < 1.

Figure 2 shows the solutions for *S*(*t*), *E*(*t*), *I*(*t*), *Q*(*t*), and *R*(*t*) obtained by NSFD, RK4, and ode45 for *ℜ*_0_ > 1, which show that it is unstable and will never become stable because of contact rates of infected people to susceptible people. So according to the act of Governments to keeping people within specified bounds (may be their home (stay at home and save your lives), offices, etc.), the contact rate will be controlled and so the current pandemic; otherwise, no control is possible. For *ℜ*_0_ < 1, when the contact rate becomes smaller, then, the current infectious disease may be controlled (see Figure 3).

## 5. Conclusion

In this work, we presented that isolation of the infected human overall can reduce the risk of future COVID-19 spread. Our model shows that the coronavirus spreads through contact and describes how fast something changes by counting the number of people who are infected and the likelihood of new infections. Those new infections are what induce the epidemic. For this reason, we think that this research may lead to better guessing of the spread of this pandemic in the future. This paper is devoted to implement the coronavirus mathematical model containing isolation class. The reproductive number-related stability is discussed, which showed the impact of interaction of infected people to susceptible population and proved graphically and analytically that if we control this contact rate, the control of the current disease is possible, otherwise. State and territory governments have different restrictions in place for public gatherings. Therefore, citizens need to follow the directions from time to time to minimize the health risk.

## Figures and Tables

**Table 1:** Parameters and description.

Symbols	Description
*S*	Susceptible population
*E*	Exposed population
*I*	Infected population
*Q*	Isolated population
*R*	Recovered population
*β*	Rate at which susceptible population moves to infected and exposed class
*π*	Rate at which exposed population moves to infected one
*γ*	Presents the rate at which exposed people take onside as isolated
*σ*	Shows the rate at which infected people were added to isolated individual
*θ*	Rate at which isolated persons recovered
*μ*	Natural death rate plus disease-related death rate

## Data Availability

The authors confirm that the data supporting the findings of this study are available within the article.
